# Diagnostic Challenges and Solutions in Systemic Amyloidosis

**DOI:** 10.3390/ijms24054655

**Published:** 2023-02-28

**Authors:** Rivka Goldis, Batia Kaplan, Olga (Lesya) Kukuy, Michael Arad, Hila Magen, Efrat Shavit-Stein, Amir Dori, Avi Livneh

**Affiliations:** 1 Department of Neurology, Sheba Medical Center, Tel Hashomer, Ramat Gan 5262000, Israel; 2 Sackler Faculty of Medicine, Tel Aviv University, Tel Aviv 6997801, Israel; 3 Institute of Hematology, Sheba Medical Center, Tel Hashomer, Ramat Gan 5262000, Israel; 4 Institute of Nephrology and Hypertension, Sheba Medical Center, Tel Hashomer, Ramat Gan 5262000, Israel; 5 Heart Failure Institute, Leviev Heart Centre, Sheba Medical Center, Tel Hashomer, Ramat Gan 5262000, Israel; 6 Multiple Myeloma Unit, Hematology Department, Sheba Medical Center, Ramat Gan 5262000, Israel; 7 Department of Medicine, Sheba Medical Center, Tel Hashomer, Ramat Gan 5262000, Israel

**Keywords:** amyloidosis, amyloid typing, free light chains, free light chain dimers, mass spectrometry, transthyretin, Western blotting

## Abstract

Amyloidosis refers to a clinically heterogeneous group of disorders characterized by the extracellular deposition of amyloid proteins in various tissues of the body. To date, 42 different amyloid proteins that originate from normal precursor proteins and are associated with distinct clinical forms of amyloidosis have been described. Identification of the amyloid type is essential in clinical practice, since prognosis and treatment regimens both vary according to the particular amyloid disease. However, typing of amyloid protein is often challenging, especially in the two most common forms of amyloidosis, i.e., the immunoglobulin light chain amyloidosis and transthyretin amyloidosis. Diagnostic methodology is based on tissue examinations as well as on noninvasive techniques including serological and imaging studies. Tissue examinations vary depending on the tissue preparation mode, i.e., whether it is fresh-frozen or fixed, and they can be carried out by ample methodologies including immunohistochemistry, immunofluorescence, immunoelectron microscopy, Western blotting, and proteomic analysis. In this review, we summarize current methodological approaches used for the diagnosis of amyloidosis and discusses their utility, advantages, and limitations. Special attention is paid to the simplicity of the procedures and their availability in clinical diagnostic laboratories. Finally, we describe new methods recently developed by our team to overcome limitations existing in the standard assays used in common practice.

## 1. Amyloidosis as a Protein Deposition Disease

Amyloidosis refers to a clinically heterogeneous group of disorders which are characterized by the misfolding of specific amyloidogenic precursor proteins that aggregate and deposit in the extracellular compartment of body tissues. Amyloidosis belongs to the protein-misfolding disorders, in which changes in protein conformation have led to self-assembly of the affected protein and formation of highly insoluble structures, called amyloid fibrils. Although all amyloid fibrils in different clinical types of amyloidosis share the same beta-pleated sheet configuration and tinctorial features, they vary with respect to the primary structure of the protein involved. Forty-two different chemical types of amyloid proteins originating from different precursor proteins have been described [1]. In immunoglobulin light chain amyloidosis (AL), found in patients with plasma cell dyscrasia (PCD), monoclonal immunoglobulin light chains are the precursor proteins of the amyloid deposits. In amyloidosis A (AA), found in patients with various forms of chronic inflammation, infections, and certain malignancies, the precursor is the acute-phase reactant, (apo)serum amyloid A (SAA). Transthyretin (TTR), a vitamin A and thyroxin carrier protein which is produced mostly by the liver, is the amyloid precursor of patients with late onset wild-type amyloidosis (ATTRwt) as well as patients with variant-/hereditary-type amyloidosis (ATTRv). AL, AA, and ATTR are the most common types of amyloidosis accounting for about 98% of all types of amyloid diseases.

Fibrillar deposits in AL amyloidosis are composed of either intact monoclonal immunoglobulin light chains, or more frequently, their fragments [2]. Similarly, in ATTR amyloidosis, the fibrils comprise either full-length TTR or a mixture of full-length TTR with fragmented TTR [3]. Truncated forms of TTR are found particularly in the heart, and an abundance of such fragments is associated with a more severe cardiac disease [4]. Amyloid fibrils in AA amyloidosis are composed of the N-terminal fragment of SAA. However, the mechanisms underlying the formation of the amyloid fibrils, the in vivo timing of the proteolytic events, and organ tropism are still poorly understood [5,6,7,8,9,10]. While some studies have suggested that amyloid precursor fragments are present in the circulation, other studies have not detected significant amounts of cleaved species in the serum, suggesting that fragmentation of the precursor proteins may occur within the tissues [11].

## 2. Clinical Manifestations of AL, ATTR, and AA Amyloidosis

Clinical symptoms are likely to be determined by the tissue involved, and there is considerable clinical overlap between all types of amyloidosis [12]. Furthermore, clinical symptoms in amyloid diseases may mimic those of more common disorders, such as diabetes mellitus, arterial hypertension, and other components of the metabolic syndrome, in which similar target organs are affected, namely small vessels, nerves, eyes, and internal organs (gastrointestinal tract, liver, kidneys, and heart). In AA amyloidosis, proteinuria leading to nephrotic syndrome and renal failure is the earliest and most frequent clinical manifestation of the disease. The gastrointestinal tract may also be affected in AA, causing malabsorption, intestinal pseudo-obstruction, diarrhea, or bleeding. ATTRwt patients present with heart failure, manifested with dyspnea on exertion, edema, and with palpitations due to atrial fibrillation, or with heart block or stroke [13]. In ATTRv amyloidosis, the specific mutation determines whether the peripheral nervous system or the heart will be predominantly or initially involved [14]. Clinical manifestations in AL amyloidosis are extremely heterogeneous. AL patients suffer from fatigue, weight loss, orthostatic hypotension, signs of progressive heart failure, arrhythmia, and thromboembolism. Some of the characteristic patterns of organ involvement in AL are indistinguishable from those of ATTR.

## 3. Importance of Determining the Amyloid Type in Clinical Practice

Identification of the amyloid type is essential in clinical practice, since both prognosis and treatment regimens are different in various amyloid disorders. For example, AL amyloidosis, but not other types of amyloidosis, may be treated by using proteasome inhibitors (bortezomib, ixazomib, and carfilzomib), immunomodulatory agents (thalidomide and lenalidomide), monoclonal antibodies (daratumumab), and in suitable patients, even by autologous bone marrow transplantation. In AA amyloidosis, the underlying inflammatory disease is targeted to prevent further generation of SAA using, for instance, colchicine and IL-1 blocking agents (anakinra or canakinumab) for FMF, or antibiotics for chronic infections, or TNF blockers for inflammatory bowel disease. In ATTRv or ATTRwt, agents arresting the production of transthyretin or stabilizing its tetramer are being used [15]. In all types of amyloidosis, early diagnosis and initiation of the appropriate treatment may attenuate or even prevent disease progression and reduce mortality.

## 4. Biopsy-Based Diagnosis of Amyloidosis: Pitfalls and Challenges

Since the clinical manifestations of different forms of amyloidosis may overlap, a diagnosis based exclusively on the clinical picture may be unreliable and should be avoided. Examination of tissue samples obtained by biopsy from an organ suspected of involvement and/or from the bone marrow is mandatory (1) to confirm the presence of amyloid fibrils and (2) to determine the type of amyloid protein composing the fibril.

Congo red (CR) staining is the most commonly used method for detecting amyloid deposits that appear dark red under bright-field microscopy and show apple-green birefringence under polarized light. New staining methods using fluorescent oligothiophenes have been recently introduced [16,17]. However, amyloid deposition is characteristically patchy, often rendering the tissue sample devoid of amyloid deposits, thereby leading to false negative results. Additional difficulties may arise from the selection of the biopsy site, which is based on the expected yield, accessibility of the site, and the risks associated with the procedure. In safer, less invasive biopsy sites (such as abdominal fat or skin), amyloid deposits may be absent or scarce and, in such cases, the diagnosis of amyloidosis is especially difficult. In view of these difficulties, it is worth mentioning the nonfibrillar amyloid-associated proteins that are regularly found to be attached to the fibrils [18,19,20,21,22]. The most well studied are serum amyloid P component (SAP), apo E, and heparan sulphate proteoglycans (HSPG); other amyloid-associated proteins, including apo A-IV, are under continuing investigation. Identification of such a “proteomic amyloid signature” (including serum amyloid P component (SAP), ApoE, and ApoA-IV) may be a more sensitive method for the diagnosis of amyloidosis than CR staining [19].

Determining the amyloid type in a CR-positive tissue can be performed using immunohistochemistry (IHC), immunofluorescence (IF), immunoelectron microscopy (IEM), and proteomic analysis [23]. IHC-based amyloid typing is used in most medical centers by employing commercial antibodies to the precursor proteins of the most common forms of amyloidosis, i.e., antibodies to κ and λ light chains, SAA, and TTR. Unfortunately, this diagnostic method is not always effective, mostly with respect to the diagnosis of AL amyloidosis [24,25,26,27,28]. This may lead to a misdiagnosis, resulting in a lack of appropriate treatment. False positive results may be obtained due to nonspecific immunostaining, caused by tissue contamination with intravascular remnant serum immunoglobulins. Conversely, false negative results may be created by poor recognition of light chains using commercial anti-κ and anti-λ antibodies due to the heterogeneity of their variable domains and truncation at the constant region [24,26]. It has been suggested that the use of more specific antibodies against light chain amyloid fibrils (not against their precursors) may overcome these limitations.

The application of IEM can significantly increase the specificity of amyloid typing [29]. The extreme microscopic magnification allows visualization of the amyloid fibrils, and following immunostaining with gold-labeled specific antibodies, it can be determined whether the antibodies bind specifically to the amyloid fibrils, or nonspecifically to extracellular non-amyloid proteins. Nevertheless, this amyloid typing method does not resolve all the flaws that might result from the insufficiently specific antibodies, as discussed above. In addition, this methodology is expensive, and not routinely used in clinical practice.

Laser microdissection of amyloid fibrils followed by liquid chromatography–tandem mass spectrometry (LC-MS/MS) is now regarded to be the “gold standard” of amyloid typing. CR-positive areas in biopsied tissue are excised, and then the proteins are digested into peptides. The resulting peptides are subjected to LC-MS/MS to identify the amino acid sequence of the deposited amyloid protein [18,29,30]. However, this method requires expensive instrumentation and highly trained personnel, and thus it is available in very few centers worldwide.

## 5. Common Noninvasive Laboratory and Imaging Techniques as Auxiliary Tools in Amyloidosis Diagnosis

An indication for the existence of either AL or ATTR may be obtained, to some extent, by noninvasive techniques (blood tests, cardiac evaluation, and digestive system/neurological examinations). These include immunofixation electrophoresis, free light chain (FLC) assay, echocardiography with strain imaging, cardiac magnetic resonance, electrocardiography, and B type natriuretic peptide and troponin in the serum. Bone scintigraphy imaging with specific tracers is often used for the diagnosis of ATTR cardiac amyloidosis, but cardiac uptake may also occur in AL.

The importance of these methods is undisputable, although the diagnostic limitations of these techniques are now well recognized. For example, detection and quantification of a monoclonal protein (the precursor of amyloid deposits in AL) using serum protein electrophoresis and immunofixation suggest PCD, but not necessarily imply AL amyloidosis. Moreover, the level of the monoclonal protein in AL amyloidosis may be too low for detection by these techniques [31,32]. Further, monoclonal immunoglobulins may be incidentally found in other types of amyloidosis, such as ATTR or AA, though without participation in amyloid fibril formation [33]. Most importantly, routine laboratory methods cannot determine the pathogenicity of the monoclonal fraction, i.e., to differentiate between the pathogenic forms of PCD (including AL amyloidosis) and the benign forms, such as monoclonal gammopathy of unknown significance (MGUS).

The potential diagnostic pitfalls which may occur when employing bone scintigraphy should also be taken into consideration. The ATTR type of amyloidosis may be suspected based on a positive cardiac uptake on a bone scan with technetium-99m-labeled tracers such as diphosphono-propanodicarboxylic acid (TcDPD), pyrophosphate, and hydroxy-methane-diphosphonate [34]. However, this technique may give false positive and false negative results. Some patients with AL amyloidosis may demonstrate a similar positive uptake; therefore, the absence of monoclonal gammopathy is needed to support a diagnosis of ATTR. Importantly, a false negative scan may occur with certain TTR mutations, one of which is the S77Y mutation [35], which is a founder mutation among Yemenite Jewish descendants and the most common cause of familial amyloidosis in Israel.

Taken together, the commonly used noninvasive techniques are helpful for determining the amyloid type in some but not all patients. Further development of reliable noninvasive tests is of a high practical value for the diagnosis and typing of amyloidosis.

## 6. Other Tests That Determine Amyloid Type

In view of the above-mentioned challenges and difficulties in the diagnosis of amyloidosis, and looking for more practical diagnostic solutions, the following tests based on analysis of serum and biopsy specimens have been developed by our team. We apply these tests to clarify the diagnosis in cases with diagnostic uncertainties, including those arising using routine IHC methods.

### 6.1. Immunochemical/Chemical Amyloid Typing in Biopsy Tissue Extracts

**Figure 1:**
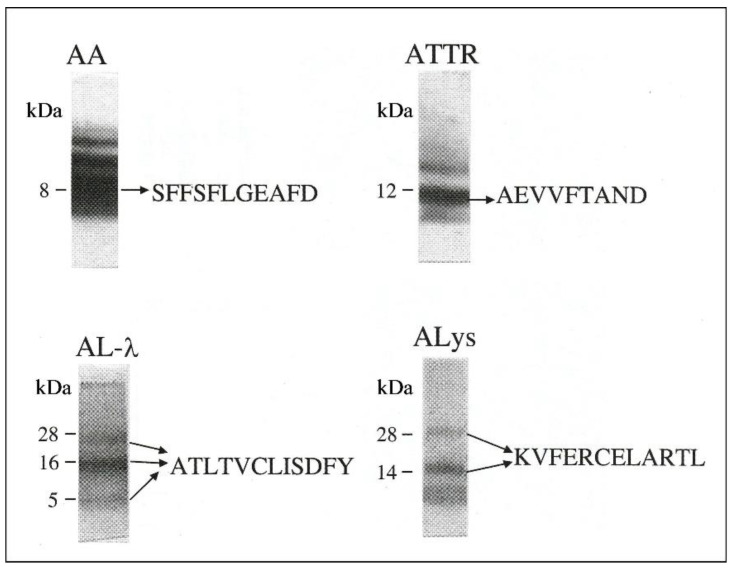
SDS-PAGE-based N-terminal amino acid sequence analysis of amyloid proteins extracted from biopsy tissues samples from 4 amyloidosis patients. N-terminal sequencing analysis was performed using the Edman degradation method. The amino acid sequences matched the amyloid type. (Currently we employ LC-MS/MS for amino acid sequencing.) AA—amyloidosis A; AL-λ—light chain lambda type amyloidosis; ATTR—amyloidosis of TTR; Alys—amyloidosis of lysozyme; LC-MS/MS—liquid chromatography–tandem mass spectrometry SDS-PAGE—sodium dodecyl sulphate—polyacrylamide gel electrophoresis (from Figure 3 in [41], by permission from Oxford University Press).

This method employs extraction of amyloid proteins from fresh-frozen or formalin-fixed biopsy specimens obtained from different tissues and organs (such as kidney, liver, gastrointestinal tract, heart, abdominal fat, and skin) [36,37,38,39,40,41,42,43,44,45,46,47,48]. Amyloid proteins are extracted from tissues using volatile solvents: aqueous 20% acetonitrile in 0.1% trifluoroacetic acid (in the case of fresh-frozen tissues), and formic acid (for formalin-fixed tissues). These solvents are then removed by Speedvac evaporation and by lyophilization. Recovery of amyloid proteins from a tissue sample is significantly increased by repeating the extraction step several times. The material extracted by these solvents has been shown to contain the major amyloid proteins, which characterize the various amyloid types: the 8 kDa amyloid A in AA amyloidosis, the 12–20 kDa light chain fragments in AL, or the TTR monomers (14 kDa) and their fragments in ATTR. Extracted proteins are separated by sodium dodecyl sulphate-polyacrylamide gel electrophoresis (SDS-PAGE). Since the major fibril components of the common amyloid diseases are relatively small proteins with molecular weights (MWs) ≤ 30 kDa, only the protein bands with MWs ≤ 30 kDa are subjected to further analysis: (a) SDS-PAGE-based Western blotting (WB) is applied for amyloid typing, using a panel of commercial antibodies against the precursors of amyloid proteins of common amyloid diseases. Then, the amyloid type is determined according to the specific immunoreactivity and MW of the detected protein. (b) To confirm the amyloid type and to determine the chemical nature of the amyloid deposit, the extracted and electrophoretically separated proteins are subjected to amino acid sequence analysis (Figure 1). For this purpose, we currently employ the LC-MS/MS on the excised and trypsin digested protein bands (MW ≤ 30 kDa). The obtained MS data are then analyzed using the Proteome Discoverer 2.4 software (Fisher Scientific) against human proteome and the unreviewed sequences of the light chains from the UniProt Knowledgebase, with 1% false discovery rate. The identified sequences are searched for those known to match a particular amyloid type.

At present LC-MS/MS (b) is being used for validation of the SDS-PAGE-based WB results. Practically, our experience shows that, in most instances, the SDS-PAGE-based WB technique provides clear-cut and reliable amyloid typing results, thus serving as an alternative to a more complex and expensive proteomic-based analysis. The usefulness of SDS-PAGE-based WB for ATTR, AL, and AA amyloid typing is illustrated in the following cases:

Patient #1

The patient was an 84 year-old male with an indolent course of dyspnea. Scintigraphy with TcDPD showed a Perugini grade 3 cardiac uptake, suggestive of ATTRwt. However, the nephelometric FLC assay showed markedly elevated κ FLC with an abnormal κ/λ ratio, suggesting a diagnosis of PCD. Thus, the type of the patient amyloidosis, i.e., AL or ATTR, remained undetermined. A WB analysis of extracted cardiac biopsy tissue demonstrated a strong 15 kDa band reactive to anti-TTR, but not to anti-κ or anti-λ antibodies. The immunoreactivity of this protein band, as well as its MW (characteristic of TTR monomer), supported the diagnosis of ATTR amyloidosis. An LC-MS/MS analysis of the 15 kDa protein-in gel band showed amino acid sequences characteristic of TTR, thus confirming the identity of the TTR-immunoreactive protein revealed by WB.

Patient #2

This patient had clinically suspected AL amyloidosis. His liver biopsy was CR positive, but the results of IHC typing were inconclusive. A WB analysis of the tissue extracted proteins showed a strong band of ~15 kDa with immunoreactivity to anti-κ, but not to anti-λ light chain antibodies. An LC-MS/MS analysis of the 15 kDa protein identified abundant sequences of the κ-light chain constant and variable regions. These findings support the diagnosis of AL-κ amyloidosis.

Patient #3

This patient, with a background of a silent Crohn’s disease and MGUS, developed cardiac amyloidosis. IHC and WB analysis of extracted cardiac tissue both showed immunoreactivity to anti-AA but not to anti-κ or anti-λ antibodies, thus indicating AA amyloidosis. This led to re-evaluation of his Crohn’s disease, which identified active inflammation, resulting in a change in his management.

The diagnostic validity of our WB technique was demonstrated by analysis of the same biopsy specimens using IF and IEM, as well as by amino acid sequence analysis of the deposited amyloid protein (Table 1). The obtained results showed that, in 40 of 41 cases, WB-based amyloid typing matched those obtained using the traditional techniques [36,37,38,39,40,41,42,43,44,47,48]. Although WB-based amyloid typing is limited to only known amyloid proteins, this technique is less expensive and more accessible compared to the “gold standard” amyloid typing technique. First, it includes a simple pre-analytical procedure, replacing the laborious laser micro-dissection of amyloid fibrils. Further, it provides information on the MW of the deposited light chains, which is another important feature of amyloid disease [10,49]. In addition, WB-based amyloid typing overcomes the limitations of routine IHC by eliminating the nonspecific signals created by tissue contamination with serum immunoglobulins: The latter are effectively separated from the lower MW amyloid proteins during the electrophoretic run. In contrast to IHC, our experience using the WB-based amyloid typing showed the utility of the commonly used commercial antibodies which allowed detection of not only intact light chains, but also their truncated species. In fact, we found that truncation of light chains was not a limitation in the WB-based amyloid typing, but rather an advantage, appearing as a useful marker of AL disease.

### 6.2. Serum FLC Monomer-Dimer Pattern Analysis in the Diagnosis of AL Amyloidosis

In some situations, a tissue biopsy is not available for evaluation, and during a long-term follow-up of as MGUS patient, repeated tissue biopsy is avoided. Therefore, we developed a noninvasive, serum-based laboratory test to support the diagnosis of AL amyloidosis [47,50,51,52,53]. In this test, we use WB to semi-quantitatively estimate the levels of two molecular forms of serum free light chains (FLC), i.e., monomers (25 kDa) and dimers (50 kDa). Namely, we measure the intensity of immunoreactive monomeric or dimeric FLC bands; the values obtained in the tested samples are normalized using a reference sample (a mixture of serum samples of healthy individuals) which is included in the same electrophoretic run. Numerical diagnostic criteria to support or reject AL amyloidosis are established by taking into consideration the monoclonal FLC (κ or λ) dimer/monomer ratio values, as well as κ/λ ratio values of dimeric FLCs and κ/λ ratio values of monomeric FLCs [50,51,52].

**Figure 2:**
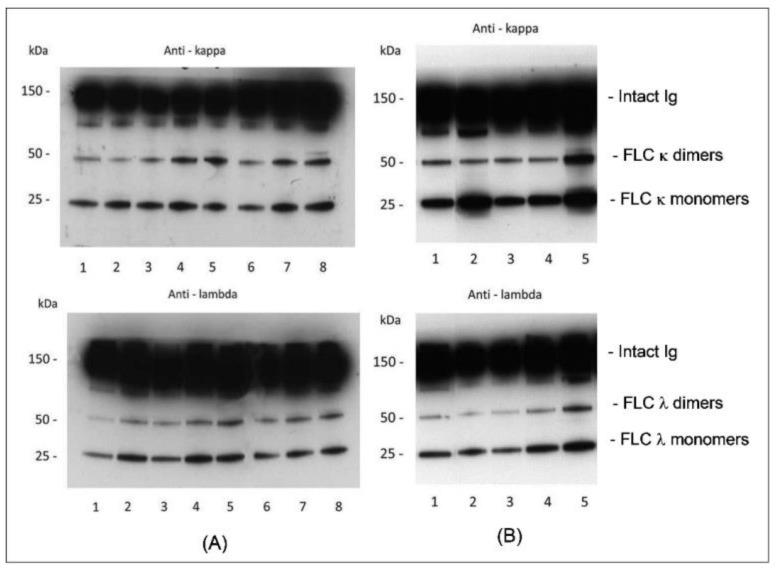
Serum FLC monomer-dimer patterns in healthy state (blot (**A**)) and MGUS (blot (**B**)). Serum samples were run using SDS-PAGE under non-reducing conditions on 10–20% Tris-glycine gels and immunoreacted with antibodies to κ and λ light chains. Bands of 25 and 50 kDa represent FLC monomers and dimers, respectively. Blot (**A**) displays the FLC monomer-dimer patterns of sera from 7 healthy subjects (Tracks 2–8). Blot (**B**) demonstrates monomer-dimer patterns of sera from 4 MGUS patients (Tracks 2–5). A reference sample (Track 1) composed of a mixture of serum samples from 15 healthy subjects was run alongside the tested samples (blots (**A**,**B**)). Compared to healthy subjects, patients with MGUS showed either normal FLC monomer-dimer patterns or increased levels of the monomeric FLC. FLC—free light chains; MGUS—monoclonal gammopathy of unknown significance; SDS-PAGE—sodium dodecyl sulphate–polyacrylamide gel electrophoresis (from Figures 2 and 4 in [51], by permission from John Willey and Sons).

**Figure 3:**
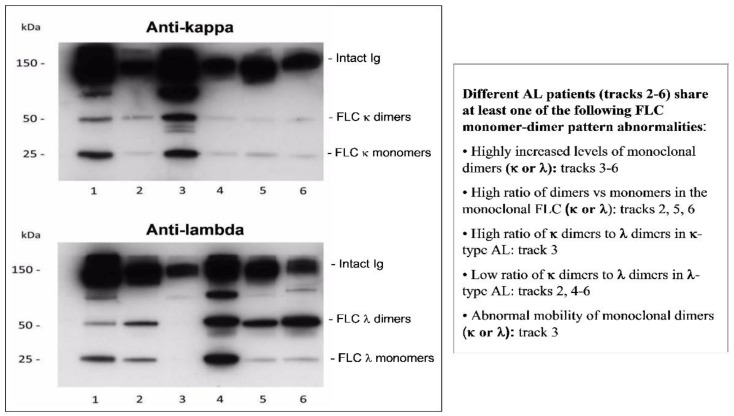
Serum FLC monomer-dimer patterns in AL amyloidosis. Serum samples were run using SDS-PAGE under non-reducing conditions on 10–20% Tris-glycine gels and immunoreacted with antibodies to κ and λ light chains. Bands of 25 and 50 kDa represent FLC monomers and dimers, respectively. Track 1—serum reference sample composed of a mixture of serum samples from 15 healthy subjects. Serum FLC monomer-dimer patterns in AL amyloidosis (Tracks 2–6) differ significantly from that in a healthy state in at least one feature delineated in the adjacent textbox: increased levels of monoclonal FLC dimers, increased monoclonal dimer/monomer ratio, and aberrant κ/λ ratio of dimeric FLC. AL—light chain amyloidosis; FLC—free light chains; SDS-PAGE—sodium dodecyl sulphate–polyacrylamide gel electrophoresis (from Figure 1 in [51], by permission from John Willey and Sons).

Figure 2 and Figure 3 demonstrate WB-based FLC monomer-dimer pattern analysis in healthy subjects, patients with MGUS, and AL patients. Compared to healthy subjects (Figure 2A), patients with MGUS (Figure 2B) showed either normal FLC monomer-dimer patterns or increased levels of the monomeric FLCs. In contrast, AL patients (Figure 3) demonstrated abnormally increased levels of monoclonal FLC dimers, invariably associated with aberrant κ/λ ratios of dimeric FLCs, which were higher than normal in AL-κ and lower than normal in AL-λ. The diagnostic utility of the noninvasive FLC monomer-dimer pattern analysis was challenged in a blinded study comparing serum samples of untreated patients with a definite diagnosis of AL and those with MGUS. Among 37 tested AL patients, 34 patients met the established FLC monomer-dimer criteria for AL, while among 23 of 26 tested MGUS patients these criteria rejected an AL diagnosis (sensitivity 92%, specificity 88.4%) [52].

In addition, FLC monomer-dimer pattern analysis enabled monitoring disease activity during the treatment course and correlated with the response to treatment. This was particularly beneficial in cases with low levels of monoclonal protein, where routine clinical data and serological tests are less informative, as illustrated in the following case of a 58-year-old AL patient, who was treated with lenalidomide, and her FLC-κ level decreased to 37.6 mg/ dL within five months. However, an increase in proteinuria (30 g/24 h) and in serum creatinine (from 0.9 to 1.63 mg/dL) were observed. Autologous stem cell transplantation (ASCT) was performed and resulted in marked improvement with a decrease in the proteinuria to 11 g/24 h and an increase in serum albumin from 2.5 to 3.4 g/dL. However, serum creatinine levels increased (2.56 mg/dL) and the urinary protein, FLC-κ, and the κ/λ ratio remained abnormal. Because the efficacy of the treatment was uncertain, an FLC dimerization test was applied for the patient follow-up. Gradual normalization of FLC patterns over the following 27 months was observed, confirming that the patient’s disease was gradually ameliorating toward a complete hematological response [50].

Further validation of the monomer-dimer pattern analysis was performed by analyzing serum samples of 10 untreated AL patients for whom the diagnosis was confirmed by biopsy examination employing the “gold standard” technique [53]. In nine of these patients, increased dimerization of monoclonal FLC was clearly observed, and the results of the FLC monomer-dimer pattern analysis supported the diagnosis of AL amyloidosis. The constant region sequences of the circulating monoclonal dimers (either κ or λ) were identified by MS, and they matched those of deposited light chain fibrils revealed by the “gold standard”-based biopsy examination. Most importantly, a remarkable sequence homology was found by comparing the variable light chain region of circulating dimers and the light chain deposits. This finding further supported the validity of the serum FLC monomer-dimer test in the diagnosis of AL amyloidosis. This test, if further substantiated across various light chain amyloidogenic sequences, can be used as a noninvasive test to confirm the diagnosis of AL amyloidosis, when a tissue biopsy is not available or when the results of a biopsy examination are negative/inconclusive but the index of suspicion remains high.

The reasons leading to increased dimerization of FLC are yet to be determined. Formation of dimeric light chains may lead to protein stabilization, and thus inhibit fibril formation [54]. Increased levels of disulfide bound FLC dimers detected in serum of AL patients may be associated with the elevated oxidative stress markers in AL [55,56]. Actually, a shift in redox potential may affect the subtle balance between dithiol and disulfide states. It is also worth mentioning that the control of disulfide bond formation is part of the quality control machinery within the cell. This is of special interest in protein-misfolding diseases, such as AL amyloidosis. Accumulation of misfolded proteins may trigger unfolded/misfolded protein responses and lead to an upregulation of folding catalysts, including oxidoreductases. The latter could stabilize the conformation of misfolded FLC by introducing disulfide bonds [57,58].

## 7. Conclusions

Amyloidosis is a rare disease with elusive presentation and prognosis which depends on the amyloid type and timely treatment. There is a high rate of diagnosis delay [59,60], which undoubtedly leads to adverse outcomes. IHC is commonly used for amyloid typing, sometimes with nonspecific findings. A WB analysis of proteins extracted from tissue biopsies allows reliable identification of the amyloid type in most common clinical forms of amyloidosis. This method has been verified using established diagnostic techniques, including MS analysis. A dimer-monomer analysis of serum FLCs may support an AL diagnosis, and the recently demonstrated sequence homology of circulating and tissue-deposited FLCs further supports the diagnostic utility of this noninvasive test. Increased awareness of these existing tests may accelerate diagnosis and expedite initiation of appropriate treatment.

## Figures and Tables

**Table 1:** Validity of WB-based amyloid typing in biopsies: A comparison with other diagnostic methods.

Amyloid Typing Techniques	Number of Cases with Identical Results
WB vs. IF	21 of 22 [36,37,39,40]
WB vs. amino acid sequence analysis of immunoreactive bands: (a) N-terminal sequencing (b) LC-MS/MS	13 of 13 [37,38,40,41,42,43,44] 6 of 6 [47,48]
	Total: 40 of 41

IF—immunofluorescence; LC-MS/MS—liquid chromatography–tandem mass spectrometry; WB—Western blotting.

## Abbreviations

AA	amyloidosis A;
AL	immunoglobulin light chain amyloidosis;
ATTR	transthyretin amyloidosis;
ATTRv	variant/hereditary type transthyretin amyloidosis
ATTRwt	Wild-type transthyretin amyloidois;
CR	Congo red;
FMF	familial Mediterranean fever;
IEM	immunoelectron microscopy;
IF	immunofluorescence;
IHC	immunohistochemistry;
LC-MS/MS	liquid chromatography–tandem mass spectrometry;
MGUS	monoclonal gammopathy of unknown significance;
MS	mass spectrometry;
MW	molecular weight;
PCD	plasma cell dyscrasia;
SAA	serum amyloid A;
SAP	serum amyloid P component;
SDS-PAGE	sodium dodecyl sulfate–polyacrylamide gel electrophoresis;
TcDPD	technetium diphosphono-propanodicarboxylic acid
TTR	transthyretin;
WB	Western blotting.
